# Extraction of first primary molars and significance of space loss: a systematic review and meta-analysis

**DOI:** 10.1186/s12903-026-07934-2

**Published:** 2026-03-13

**Authors:** Ahmed Kamal El-Motayam, Mostafa A. Hassan, Mais Medhat Sadek, Rim Fathalla, Nour Wahba, Reem Mahmoud, Shereen Hassan Elshamy

**Affiliations:** 1 Department of Pediatric Dentistry and Dental Public Health, Faculty of Dentistry, Cairo University, 11 Al Saraya street, Manial, Cairo, Egypt; 2 Royal Dentists Institute, 74 Mohamed Farid Street, Cairo Nasr City, Egypt; 3 University of Sharjah,Ain Shams University, P. O. Box 82828, Cairo, Egypt; 4 Faculty of Dentistry, Suez Canal University, 4.5 km ring road, Ismailia, Egypt; 5 Faculty of Dentistry, Ain Shams University, El-Khalifa El-Mamoun Street, Cairo, Egypt; 6 Faculty of Dentistry, British University in Egypt, Suez Desert Road, P.O. Box 43, Cairo El Sherouk City, 11837 Egypt

**Keywords:** Space loss, Premature tooth loss, Primary first molar, Spatial changes, Space maintainer

## Abstract

**Objective:**

To quantify space loss following premature extraction of primary first molars (Ds) over a follow-up period of at least six months and evaluate its clinical implications.

**Materials and methods:**

A systematic search of PubMed, Cochrane Library, LILACS, Web of Science, and Embase was conducted. Only longitudinal split-mouth cohort studies were included. The primary outcome was the amount of space loss in the extraction region, measured against the contralateral control side.

**Results:**

7 studies were included, comprising 141 children for maxillary analysis and 171 children for mandibular analysis. Meta-analysis for space loss revealed a mean difference (MD) of -0.52 mm (95% CI: -0.79 to -0.26; *p* < 0.001) in the maxilla after 9–12 months, and − 1.78 mm (95% CI: -2.09 to -1.47; *p* < 0.001) in the mandible after 8–9 months.

**Conclusions:**

Premature loss of Ds in children aged 6–9 years results in space loss of -0.52 mm in the maxilla and − 1.78 mm in the mandible. Space maintainers are indicated following mandibular extractions, while maxillary cases may not require routine intervention.

**Supplementary Information:**

The online version contains supplementary material available at 10.1186/s12903-026-07934-2.

## Introduction

The early loss of primary teeth can significantly affect the development of permanent dentition and has long been a topic of research interest. A recent systematic review and meta-analysis indicates a significant association between the early loss of primary teeth and an increased prevalence of malocclusion in permanent dentition [1], which may manifest as increased overjet, crowding, disturbed molar relationships, or space loss [2, 3].

The amount of space loss depends on which primary molar is extracted. Primary second molars are frequently extracted prematurely [3], and significant space loss has been well documented [4–6], making space maintainers commonly recommended [4]. In contrast, the early loss of primary first molars (Ds) and the clinical significance of subsequent space loss remain controversial [2]. Given that space maintainers may increase the risk of decalcification below the bands [7], their use must be carefully considered.

The need to distinguish between maxillary and mandibular space loss arises from fundamental arch differences in both the magnitude of loss and the capacity for compensation. The leeway space—the differential in size between primary canine and molars and their successors—varies by arch and serves as a key reserve for mitigating space deficits [3, 8]. Therefore, quantifying arch-specific space loss patterns is a prerequisite for evidence-based decisions on space maintenance to prevent arch-length discrepancy and malocclusion.

Unlike prior systematic reviews that included cross-sectional data or lacked quantitative synthesis [9–11], this study presents the first meta-analysis restricted to longitudinal split-mouth designs. Our aim is to produce a robust, separate estimates of maxillary and mandibular space changes following premature extraction of Ds and to determine whether space maintenance is clinically indicated in each arch.

## Methods

This review was conducted in accordance with the Preferred Reporting Items for Systematic Reviews and Meta-Analyses (PRISMA) guidelines [12]. The study protocol was registered prospectively with PROSPERO (CRD42025640574). The review addressed the following question: “In children requiring extraction of a first primary molar, what is the magnitude of space loss compared to the contralateral non-extraction side over a follow-up period of at least six months?”

### Literature search strategy

A comprehensive search was performed in PubMed, Embase, Web of Science, Lilacs, and Cochrane Library (see Supplementary Material for full search strategy). Search terms included combinations of: (‘Premature’ OR ‘early’) AND (‘deciduous molars’ OR ‘primary molars’ OR ‘temporary molars’ OR ‘Baby molars’) AND (‘loss’ OR ‘extraction’) AND (‘Space loss’ OR ‘migration’ OR ‘drift’). Boolean operators (OR, AND) were used to combine terms. Reference lists of included studies were also screened. Search results were imported into EndNote version 20, and duplicates were removed.

### Inclusion and exclusion criteria

Studies were selected based on the PECOS framework:

- *Population*: Children aged 4–9 years.
- *Exposure*: Extraction of a first primary molar.
- *Comparison*: Contralateral non-extraction side in the same patient (split-mouth design).
- *Outcome*: the amount of change in the Ds space in millimeters.
- *Study design*: Prospective or retrospective cohort studies.

Exclusion criteria were:

- (i) duplicate publications;
- (ii) unclear study protocols;
- (iii) follow-up period <6 months;
- (iv) use of space maintainers post-extraction;
- (v) reviews, case reports, letters, or cross-sectional studies.

Titles and abstracts were screened independently by two reviewers (A.K.E., a pediatric dentist, and M.A.H., an orthodontist). Any disagreements were resolved through consultation with a third reviewer (N.W., a pediatric dentist). All reviewers were experienced in systematic review methodology.

### Data extraction

Data were extracted independently by two authors (A.K.E and M.A.H.) based on the Strengthening the Reporting of Observational Studies in Epidemiology (STROBE) recommendations [13]. Information extracted from each article included author details, year of publication, study design, sample size, tooth extracted, follow-up duration, eruption stage of the first permanent molar, and changes in D or D + E space. For clarity, D represents the first primary molar, E is the second primary molar, and D + E space is the combined space occupied by both.

To ensure all eligible studies could be included in the quantitative synthesis, data were extracted to calculate the necessary outcome measures. For studies that did not explicitly report mean differences (MD) or space loss, the values were calculated from the available data, such as baseline and final examination values, standard deviations, and sample sizes, following established meta-analysis methods [14] (Cochrane Handbook for Systematic Reviews, Sect. 10.2). This process allowed us to produce a robust pooled estimate and ensure all studies meeting our criteria were included in the meta-analysis.

### Quality assessment

The quality of the included studies was assessed using the ROBINS-I tool, developed by the Cochrane Collaboration [15]. This tool evaluates the risk of bias across seven domains: bias due to confounding, bias in the selection of participants, bias in the classification of interventions, bias from deviations from intended interventions, bias due to missing data, bias in the measurement of outcomes, and bias in the selection of reported results. Two independent reviewers (A.K.M. and M.A.H.) conducted the assessments to reduce subjective bias. Any disagreements between the two reviewers were initially resolved through discussion and consensus. If a consensus could not be reached, a pre-specified third reviewer (N.W.) was consulted to provide a final decision.

### Statistical analysis

To test statistical significance in the differences in spaces between the extraction and control sides, and space losses between baseline and final examination values, MD were calculated for D + E and D spaces. A comprehensive meta-analysis was conducted using Stata 17 software to synthesize data from multiple studies. The primary analysis employed a fixed-effects model with inverse variance weighting to calculate pooled effect estimates and 95% confidence intervals (CIs). Statistical significance was defined as *p* < 0.05. Heterogeneity among studies was assessed using the I² statistic and Cochran’s Q test. Substantial heterogeneity was considered present if I² > 50% or *p* < 0.1 for the Q test. When heterogeneity was present, we shifted to a random-effects model, specifically using the DerSimonian and Laird (DL) method. To ensure the robustness of our findings, a sensitivity analysis was performed using the leave-one-out method, which involved omitting one study at a time to observe its impact on the overall results. Additionally, potential publication bias was investigated using Egger’s test, Begg’s test, and visual inspection of a funnel plot. A p-value of less than 0.05 in either Egger’s or Begg’s test suggested significant publication bias.

## Results

### Search and selection results

**Fig. 1:**
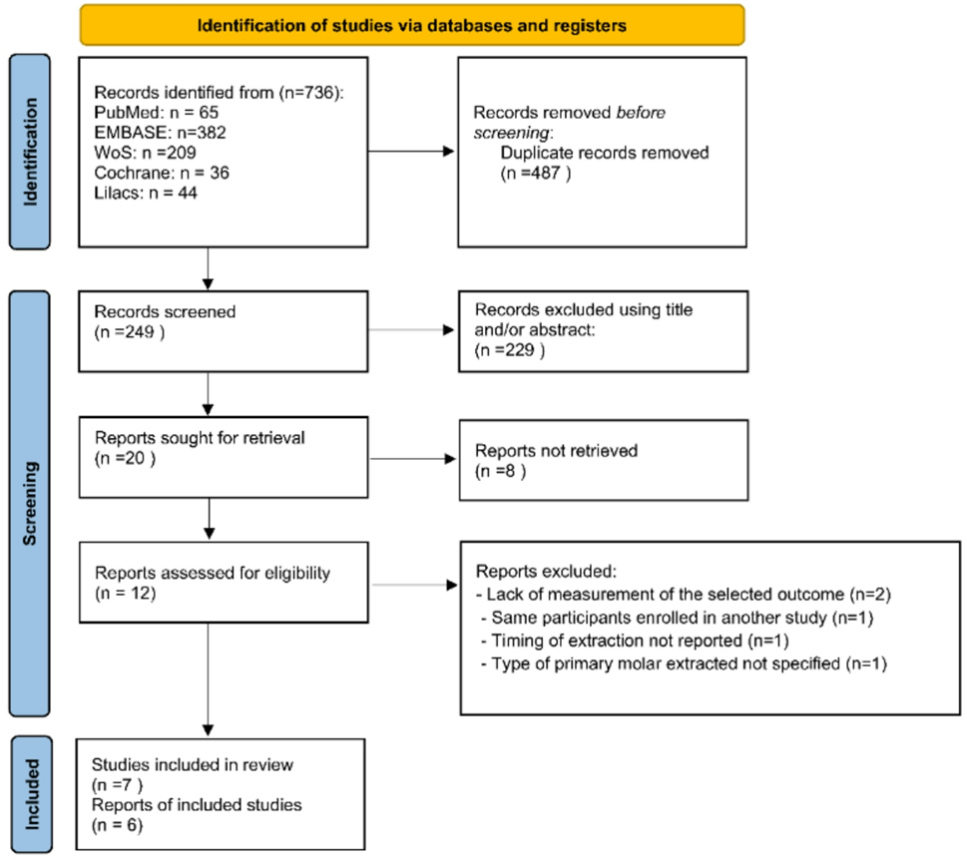
PRISMA flow diagram showing the study selection process

The systematic search, completed in June 2025, identified 736 records from five databases: PubMed (*n* = 65), Embase (*n* = 382), Web of Science (*n* = 209), Cochrane Library (*n* = 36), and LILACS (*n* = 44) (Fig. 1). After removing duplicates, 249 titles and abstracts were screened. Of the 20 studies sought for full-text review, eight were unavailable. The remaining twelve were assessed, and five were excluded due to: lack of relevant outcome data [4, 16], duplicate population [17], unrecorded extraction timing [18], or unspecified molar type [5]. Seven studies ultimately met al.l eligibility criteria and were included in the review [19–25].

### Characteristics of the included studies

The characteristics of the seven included studies are summarized in Table 1. All were prospective cohort studies utilizing a split-mouth design, comparing the extraction side to the contralateral control side. Studies were conducted in Taiwan [20, 21], India [22, 23], Korea [25], Poland [24], and the USA [19]. Sample sizes ranged from 11 [22, 25] to 226 children [19]. Follow-up periods ranged from 8 to 12 months.

**Table 1:** Characteristics of included studies

Study	Age	Sample size	Follow up period(months)	Exposure(tooth extraction)	Outcome measure	Results (mms )				Eruption state of permanent first molar
							Extraction side		Control side	
							Upper			
Kundra et al. [25]	6-9 yrs	20	9	Upper or lower D	D space	Pre Post	8.03±0.87		8.35±0.87	erupted
							6.50±0.99		8.35±0.87	
							Lower			
						PrePost	8.03±0.87	8.35±0.87		
							6.58±1.08	8.35±0.87		
Kobyliniska et al. [27]	5-7 yrs	37	12	Upper or lower D	D space	Pre Post	Upper			Erupted and unerupted
							7.156		7.531	
							6.281		7.438	
						Pre Post	Lower			
							7.929		8.250	
							6.964		7.964	
Alexander et al. [8]	7 .8 – 8.2 yrs	226	9	upper or lower D	D+E space	1^st^ group Pre Post PrePost	Upper			erupted
							16.98 ± 1.41		16.80 ± 1.28	
							15.23 ± 1.33		16.71 ± 1.36	
							­ Lower			
							17.99 ± 1.63		17.79 ± 1.41	
							16.61 ± 1.74		17.65 ± 1.30	
						2^nd^ group PrePost PrePost	Upper			
							17.15 ± 1.40		17.11 ± 1.30	
							17.22 ± 1.51		17.05 ± 1.26	
							Lower			
							17.61 ± 1.55		17.60 ± 1.38	
							16.02 ± 0.29		17.71 ± 1.42	
						3^rd^ groupPrePost PrePost	Upper			
							16.80 ± 1.66		16.91 ± 1.41	
							15.91 ± 0.43		16.81 ± 1.41	
							Lower			
							17.96 ± 1.71		17.65 ± 1.39	
							16.25 ± 0.93		17.59 ± 1.39	
						4^th^ groupPrePost PrePost	Upper			
							17.05 ± 1.22		17.11 ± 1.35	
							16.94 ± 1.20		17.20 ± 1.43	
							Lower			
							17.88 ± 1.71		17.71 ± 1.43	
							17.80 ± 1.69		17.66 ± 1.39	
Lin et al. 2011 [5]	Mean 6 years (±0.74)	19	12	upper D	D+E space	Pre Post	16.66 ± 0.94		16.93 ± 1.03	Erupted or about to erupt
							15.84 ± 0.97		16.92 ± 1.11	
Park et al. [26]	7 yrs 11 months(mean)	13	12	upper D	D+E space		16.5 ± 1.0		16.1 ± 0.8	erupted
							15.9 ± 1.2		15.8 ± 0.9	
Kumari and Retnakumari [24]	6-9 yrs	87	8	lower D	D space	Pre Post	8.23 ± 0.52		8.31 ± 0.52	erupted
							6.4 ± 0.44		8.28 ± 0.55	
Lin and Chang [10]	6yrs 11 months(mean)	21	8	lower D	D+E space	Pre Post	18.06 +1.81		17.86 +1.32	Erupted or about to erupt
							16.84 +1.86		17.83 +1.30	

### Risk of bias assessment

**Fig. 2:**
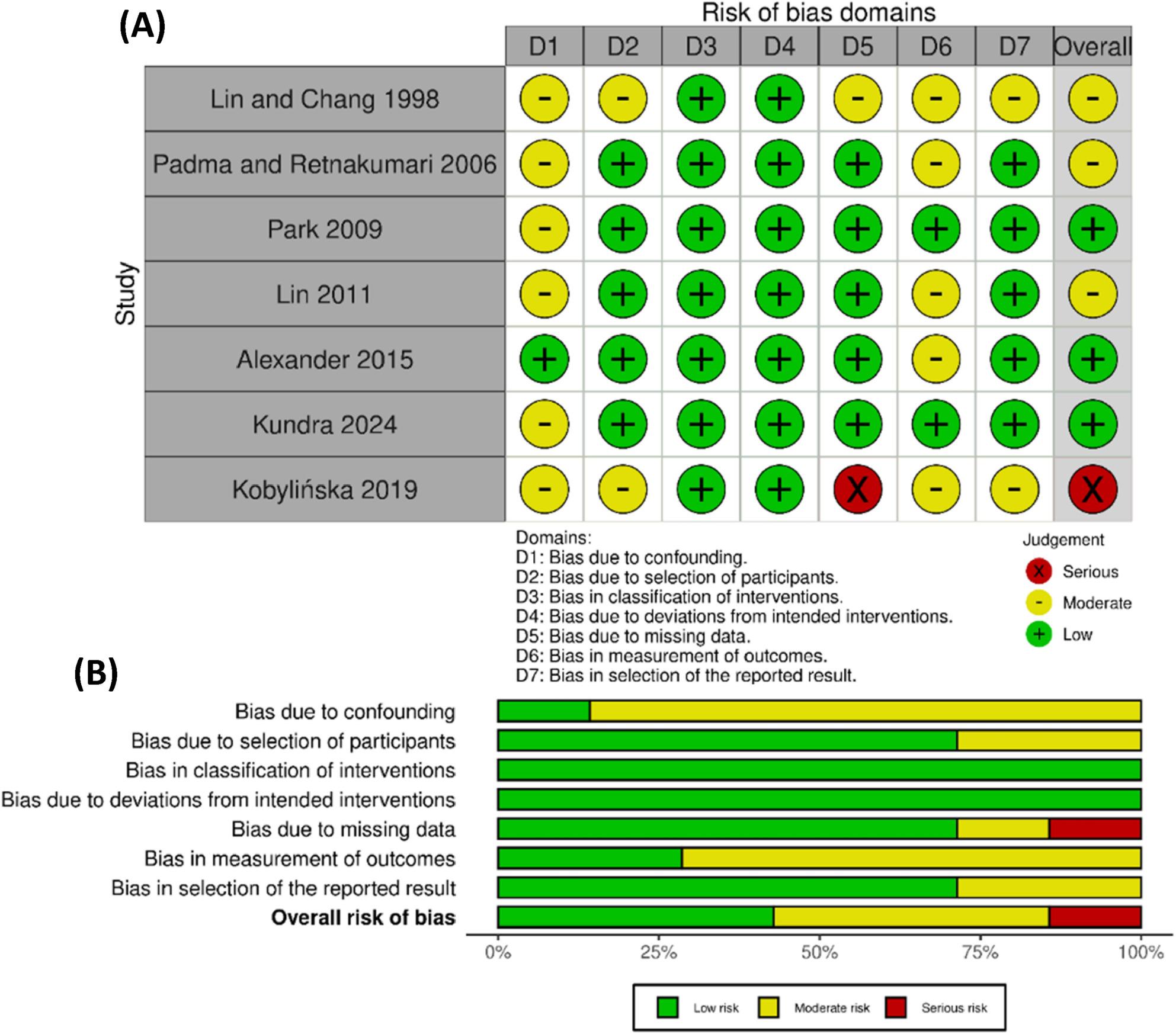
Risk of bias assessment using the ROBINS-I tool: 2 **A**, Traffic light plot for individual studies; 2 **B**, Summary plot across bias domains

Risk of bias assessment results are summarized in Fig. 2. Using the ROBINS-I tool, most studies were judged to have a moderate overall risk of bias. The most frequent methodological concerns were moderate risks due to confounding (Domain 1) and bias in the measurement of outcomes (Domain 6). Three studies were assessed as having the lowest risk of bias: Alexander et al. [19] (moderate risk only in Domain 6), Kundra et al. [22] (moderate risk only in Domain 2), and Park et al. [25] (moderate risk only in Domain 1); all other domains for these studies were low risk. In contrast, Kobylińska et al. [24] exhibited a serious risk of bias, primarily due to bias due to missing data (Domain 5). Due to these serious methodological concerns, the study was excluded from the quantitative meta-analysis.

### Quantitative synthesis and meta-analysis

Data from 141 children were included in maxillary space change analyses and 171 children for mandibular space change analyses. Results are reported as mean differences (extraction side minus control side).

### Maxillary space loss

**Fig. 3:**
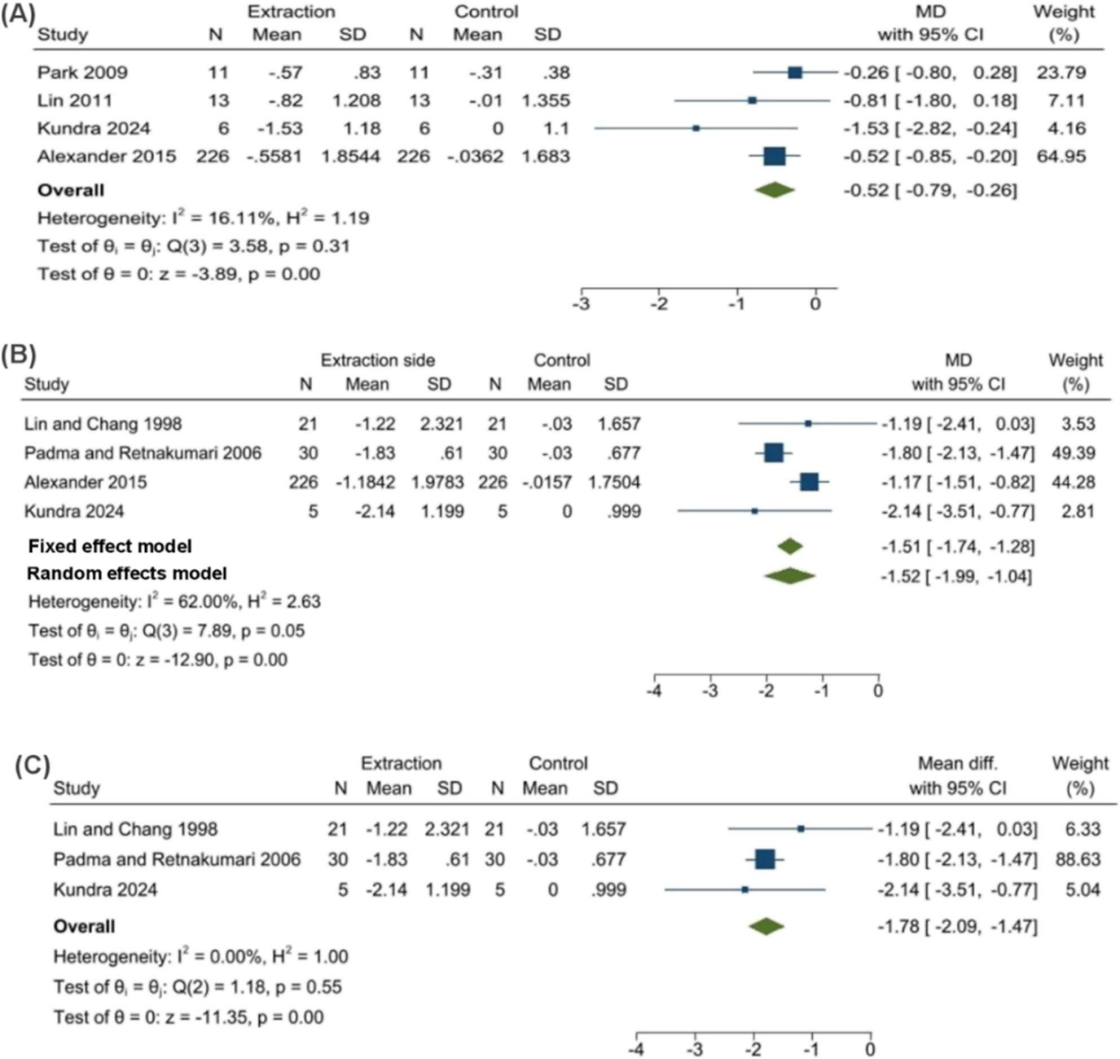
Forest plots for space changes. 3 **A**, Maxillary arch. 3 **B**, Mandibular arch (initial analysis). 3 **C**, Mandibular arch after exclusion of the Alexander study to resolve heterogeneity

**Fig. 4:**
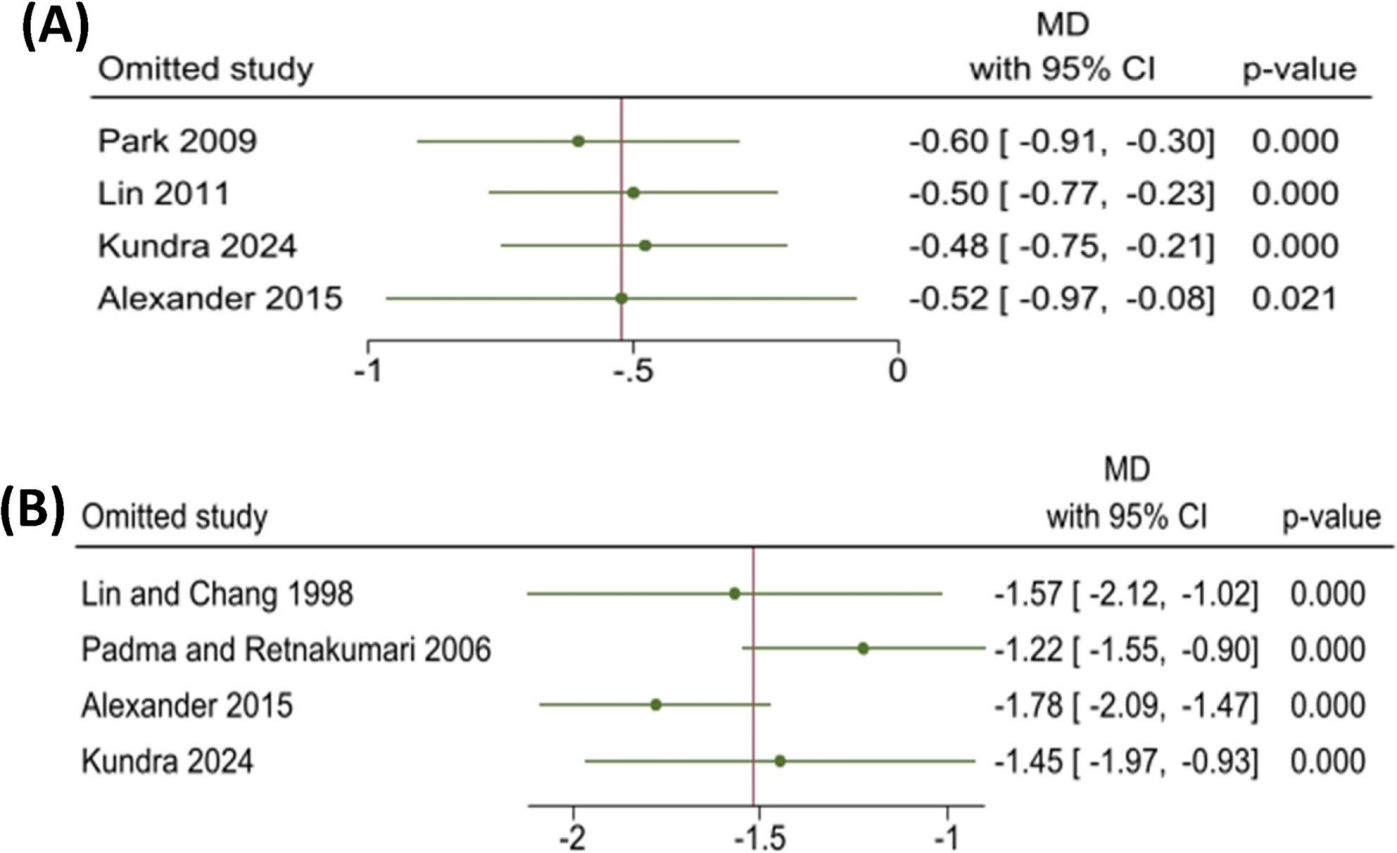
Sensitivity analysis for included studies: 4 **A**, Maxillary arch; 4 **B**, Mandibular arch

Using a fixed-effects model (Fig. 3A), the mean difference was − 0.52 mm (95% CI: -0.79, -0.26; *p* < 0.001) after 9–12 months. The analysis showed homogeneity (I² = 16.11%,Q = 3.58, *p* = 0.31). Sensitivity analysis (Fig. 4A) confirmed statistical significance after sequential exclusion of individual studies.

### Mandibular space loss

Initial fixed-effects analysis showed a mean difference of -1.51 mm (95% CI: -1.74, -1.28; *P* < 0.001) after 8–9 months, with substantial heterogeneity (I² = 62%, Q = 7.89, *p* = 0.05). Random-effects analysis yielded − 1.52 mm (95% CI: -1.99, -1.04; *p* < 0.001) (Fig. 3B). Sensitivity analysis (Fig. 4B) identified Alexander et al. [19] (which used direct intraoral measurement vs. dental models in other studies) as a source of heterogeneity. After excluding this study, heterogeneity was eliminated (I² = 0%, Q = 1.18, *p* = 0.55). Given the homogeneous data, we applied a fixed-effects model, which yielded a pooled MD of -1.78 mm (95% CI: -2.09, -1.47; *p* < 0.001) (Fig. 3C). This result was chosen as the final estimate for mandibular space loss.

### Publication bias

**Fig. 5:**
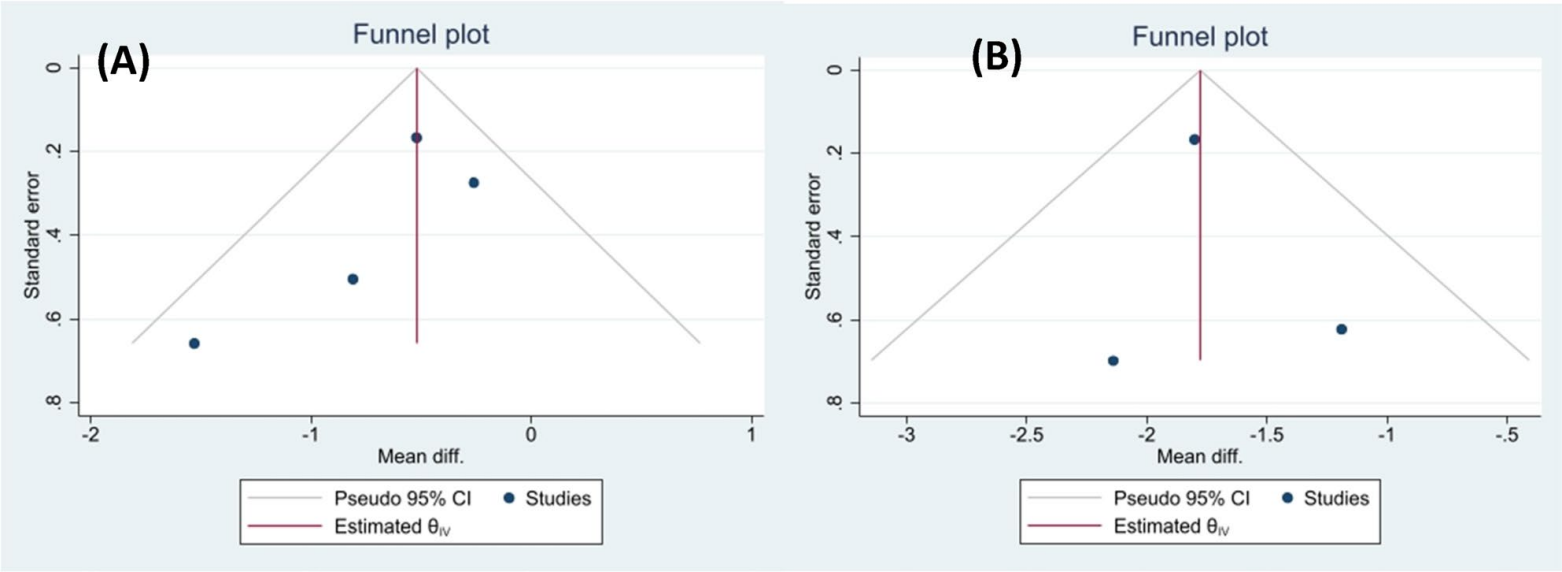
Funnel plots assessing publication bias: 5 **A**, Maxillary space loss; 5 **B**, Mandibular space loss

The results of Egger’s test (maxillary: *p* = 0.2221, mandibular: *p* = 0.7644) and Begg’s test (maxillary: *p* = 0.3082, mandibular: *p* = 1.0) indicated no evidence of publication bias. This was further supported by visual inspection of symmetrical funnel plots (Fig. 5).

## Discussion

This meta-analysis confirms that premature extraction of Ds results in space loss in both jaws, with mandibular loss substantially exceeding maxillary loss. While Park et al. [25] found no significant differences between extraction and control sides, the remaining studies reported measurable reductions in both arches [18–23].

The eruption status of the first permanent molar may influence space closure patterns. Kobylińska et al. [24] reported nearly threefold greater loss when the permanent molar was unerupted (− 1.60 mm) versus erupted (− 0.58 mm), suggesting that occlusal intercuspation after eruption may stabilize the arch. However, methodological limitations (small sample, high attrition, missing data) limit interpretation, underscoring the need for larger, well-stratified longitudinal studies to clarify the clinical significance of eruption timing on space management decisions.

### Heterogeneity and measurement reliability

Methodological consistency also directly impacted result interpretation. While maxillary analysis showed excellent homogeneity (I² = 16.11%) and was therefore analyzed using a fixed-effects model, substantial heterogeneity was initially present for mandibular outcomes (I² = 62%). This was primarily attributable to Alexander et al. [19], which utilized direct intraoral measurements rather than the dental model analysis employed by other studies. Intraoral measurements in the posterior mandible are less accurate due to access limitations and patient cooperation challenges [26]. Excluding this study eliminated heterogeneity (I² = 0%); a final fixed-effects model yielded a pooled estimate of − 1.78 mm (95% CI: -2.09, -1.47), an increase from the initial estimate of − 1.51 mm (95% CI: -1.74, -1.28). This suggests intraoral measurements may underestimate true space loss and highlights the critical need for standardized, model-based measurement protocols in future research.

Furthermore, our exclusive focus on longitudinal studies explains key differences from prior syntheses. While our maxillary estimate aligns with Zhao et al. [11], our greater mandibular loss (− 1.78 mm vs. −1.07 mm) likely reflects our ability to isolate post-extraction changes from pre-existing caries-related space loss captured in cross-sectional designs. Evidence indicates that proximal caries alone can account for up to 1 mm of space loss per quadrant before extraction [27].

### Clinical implications and space management

The necessity of space maintenance following Ds extraction remains debated. Some clinicians advocate for its use in high-risk situations, such as severe arch length discrepancy, Class II molar relationships, or hyperdivergent growth patterns [9, 19]. Others argue that the available leeway space makes space maintainers unnecessary following Ds loss [4, 25, 28]. However, space loss of exceeding 1 mm is generally regarded as clinically significant, as it is associated with detectable crowding, molar rotation, or arch-length deficiency requiring intervention [3, 9, 20].

This leeway surplus, also termed E-space [29], can be preserved with a lingual holding arch to compensate for space loss during the late mixed dentition stage [30]. While effective, this approach carries potential drawbacks: risk of enamel decalcification on the abutment molars [31], possible interference with the physiologic “late mesial shift” of the first permanent molar [32], and increased likelihood of second permanent molar impaction [33, 34].

If the normal dentitional development process of mesialization of the first permanent molar is to be left undisturbed, the only space surplus that can compensate for space loss resulting from early extraction of Ds would be the size difference between the first premolar and its predecessor. The primary first molar is larger than its successor on average by 0.35 mm (± 0.8) and 0.61 mm (± 0.8) in the maxilla and mandible respectively [35] with minor population variations [36–40].

### Maxillary space management

Based on our meta-analysis, maxillary space loss (-0.52 mm, 95% CI: -0.79, -0.26) remains below the 1 mm clinical threshold and approximates the natural compensatory capacity (0.35 mm size difference). This suggests that in many maxillary cases, natural compensation may be sufficient, potentially reducing the need for a space maintainer. However, this assumes ideal conditions and does not account for individual variability in tooth size, arch length discrepancies, or other complicating factors.

### Mandibular space management

The mandibular findings present a clearer clinical indication. With space loss averaging − 1.78 mm (95% CI: -2.09, -1.47) and available compensatory space of only 0.61 mm, a clinically significant deficit of approximately 1.2 mm remains. This magnitude of space loss substantially exceeds the 1 mm clinical threshold and the natural compensatory mechanisms, thereby strongly supporting routine use of space maintainers following mandibular Ds extraction. Given that the greatest space loss typically occurs within the first three months post-extraction [4, 41], timely placement of space maintainers is recommended to preserve arch length, guide premolar eruption, and maintain occlusal harmony.

### Limitations and future directions

This systematic review possesses several methodological strengths that enhance the reliability of our findings. The exclusive inclusion of longitudinal studies improves the quality and homogeneity of the pooled evidence by eliminating the confounding effects of pre-extraction space loss. The absence of publication bias, as demonstrated by Egger’s and Begg’s tests together with symmetrical funnel plots, further supports the robustness of our results. Additionally, comprehensive sensitivity analyses confirmed the stability of our findings.

However, important limitations must be acknowledged. The relatively small number of eligible studies (*n* = 7) limits statistical power and generalizability. Potential measurement bias arises from the synthesis of studies using mixed methods (digital models, plaster models, and intraoral measurements), compounded by the widespread absence of reported inter-examiner calibration or intra-examiner reliability. This methodological inconsistency reduces confidence in the precision of recorded measurements, as undetected variability could have influenced the reported outcomes.

Another limitation is the inability to perform formal subgroup analysis based on first permanent molar eruption status. Although Kobylińska et al. [24] provided evidence for greater space loss when permanent molars are unerupted, methodological shortcomings prevented quantitative inclusion of this potentially important factor. Furthermore, pre-extraction conditions such as caries or interproximal wear were not consistently documented, despite their potential influence on space changes.

Despite these limitations, our findings provide clinically relevant guidance: space maintainers are particularly indicated in the mandible, while their use in the maxilla may be more selective. Future longitudinal studies with standardized measurement methods, explicit reporting of examiner reliability, and careful control of pre-extraction factors are needed to refine clinical recommendations and preventive orthodontic strategies.

## Conclusion

Premature loss of Ds results in measurable space reduction in both arches: -1.78 mm (95% CI: -2.09, -1.47) in the mandible and − 0.52 mm (95% CI: -0.79, -0.26) in the maxilla. This space loss is clinically significant in the mandible and often self-compensated in the maxilla. We recommend routine space maintenance for mandibular extractions, with timely placement within three months when most space loss occurs. In contrast, maxillary space maintenance is not routinely necessary. Clinical decisions should be individualized based on existing crowding, arch integrity, and patient-specific risk factors.

## Supplementary Information

Supplementary Material 1.

## Data Availability

The datasets analyzed in this study are available from the corresponding author upon reasonable request.

## Abbreviations

ROBINS: I Risk Of Bias In Non–randomized Studies–of Interventions
