# Supplementary material for: Extraction of first primary molars and significance of space loss: a systematic review and meta-analysis
Source: BMC Oral Health. 2026 Mar 13;26:611. doi: 10.1186/s12903-026-07934-2 (PMC13063717; doi:10.1186/s12903-026-07934-2)
Supplement: Supplementary file 1 — Supplementary Material 1. [file 12903_2026_7934_MOESM1_ESM.docx]

**Search strategy**

1. PubMed , Chocrane library and Lilacs

| #1 | primary AND molar* |
| --- | --- |
| #2 | deciduous AND molar* |
| #3 | temporary AND molar* |
| #4 | baby AND molar* |
| #5 | #1 OR #2 OR #3 OR #4 |
| #6 | premature* extract* |
| #7 | premature* los* |
| #8 | early los* |
| #9 | early extract* |
| #10 | #6 OR #7 OR #8 OR #9 |
| #11 | space los* |
| #12 | spatial change* |
| #13 | space change* |
| #14 | migrat* |
| #15 | drift |
| #16 | #11 OR #12 OR #13 OR #14 OR #15 |
| #17 | #5 AND #10 AND #16 |

1. Web of Science

| #1 | TS= primary NEAR/2 molar* |
| --- | --- |
| #2 | TS= deciduous NEAR/2 molar* |
| #3 | TS= temporary NEAR/2 molar* |
| #4 | TS= baby NEAR/2 molar* |
| #5 | #1 OR #2 OR #3 OR #4 |
| #6 | TS= premature* extract* |
| #7 | TS= premature* los* |
| #8 | TS= early los* |
| #9 | TS= early extract* |
| #10 | #6 OR #7 OR #8 OR #9 |
| #11 | TS= space los* |
| #12 | TS= spatial change* |
| #13 | TS= space change* |
| #14 | TS= migrat* |
| #15 | TS= drift |
| #16 | #11 OR #12 OR #13 OR #14 OR #15 |
| #17 | #5 AND #10 AND #16 |

3.EMBASE

| #1 | (primary adj2 molar*).ti,ab,kw. |
| --- | --- |
| #2 | (deciduous adj2 molar*).ti,ab,kw. |
| #3 | (temporary adj2 molar*).ti,ab,kw. |
| #4 | (baby adj2 molar*).ti,ab,kw. |
| #5 | primary tooth/exp |
| #6 | #1 OR #2 OR #3 OR #4 OR #5 |
| #7 | (premature* adj2 extract*).ti,ab,kw. |
| #8 | (premature* adj2 los*).ti,ab,kw. |
| #9 | (early adj2 los*).ti,ab,kw. |
| #10 | (early adj2 extract*).ti,ab,kw. |
| #11 | tooth extraction/exp |
| #12 | #7 OR #8 OR #9 OR #10 OR #11 |
| #13 | (space adj2 los*).ti,ab,kw. |
| #14 | (spatial adj2 change*).ti,ab,kw. |
| #15 | (space adj2 change*).ti,ab,kw. |
| #16 | migrat*.ti,ab,kw. |
| #17 | drift.ti,ab,kw. |
| #18 | tooth migration/exp |
| #19 | #13 OR #14 OR #15 OR #16 OR #17 OR #18 |
| #20 | #6 AND #12 AND #19 |

***Explanatory Note:*** *The search syntax was adapted to the specific requirements and functionality of each database. Truncation symbols (e.g., * for PubMed, Cochrane, Embase) and proximity operators (e.g., NEAR/n, adj) were applied where supported to capture relevant variations of search terms. Boolean operators (AND, OR) were standardized across all platforms. Despite minor variations in syntax, the core search logic and term groupings remained consistent to ensure comprehensive and comparable retrieval across databases.*
